# In search of experimental evidence on Scratch programming and students’ achievements in the first-year college computing class? Consider these datasets

**DOI:** 10.1016/j.dib.2022.108635

**Published:** 2022-09-24

**Authors:** Oladele O. Campbell, Harrison I. Atagana

**Affiliations:** a Department of Computer Science, Niger State Polytechnic, Zungeru. Niger State. Nigeria; b Institute for Science and Technology Education, College of Science, Engineering and Technology, University of South Africa, South Africa; c Institute for Nanotechnology & Water Sustainability, College of Science, Engineering and Technology, University of South Africa, South Africa

**Keywords:** CS1, Novice programming, Constructionism, Block-based programming, Experimental data, Coarsened Exact Matching, Quasi-experiment, CS1, Computer Science One

## Abstract

This article presents datasets representing the demographics and achievements of computer science students in their first programming courses (CS1). They were collected from a research project comparing the effects of a constructionist Scratch programming and the conventional instructions on the achievements of CS1 students from selected Nigerian public colleges. The project consisted of two consecutive quasi-experiments. In both cases, we adopted a non-equivalent pretest-posttest control group design and multistage sampling. Institutions were selected following purposive sampling, and those selected were randomly assigned to the Scratch programming class (experimental) and the conventional (comparison) class. A questionnaire and pre- and post-introductory programming achievement tests were used to collect data. To strengthen the research design, we used the Coarsened Exact Matching (CEM) algorithm to create matched samples from the unmatched data obtained from both experiments.

Future studies can use these data to identify the factors influencing CS1 students' performance, investigate how programming pedagogies or tools affect CS1 students' achievements in higher education, identify important trends using machine learning techniques, and address additional research ideas.

**Specifications Table**

**Table:** 

Subject	Computer Science
Specific subject area	Pedagogies for teaching novice computer science students
Type of data	Table Figure
How the data were acquired	These data were collected from 4 cohorts of first-year polytechnic computer science students, representing 4 treatment groups. A CS1 student profile questionnaire, a pre- and post-Introductory Programming Achievement Tests, all paper based, were used to acquire the data. Then, the Coarsened Exact Matching (CEM) algorithm was employed to generate matched treatment samples from the data. This resulted in 2 pairs of equivalent samples from the 4 treatment groups.
Data format	Raw. Filtered. Analysed
Description of data collection	These datasets were gathered from 4 cohorts of Nigerian polytechnic CS1 students who participated in 2 successive experiments, spanning 2 academic sessions. Institutions were selected using purposive sampling, and those selected were randomly assigned to treatment groups. The data collected include student profiles and pre-post achievement test scores. The participants were administered paper-based questionnaires and achievement tests. A computer science educator marked all the achievement tests, following the rubric presented in [1]. Data were collected from 520 first-year computer science students. We excluded data from subjects who did not complete all 3 instruments, leaving data from 418 participants. Data were coded, captured and processed using Microsoft Excel and Statistics Products and Service Solution (SPSS) version 23. The Coarsened Exact Matching (CEM) program, installed as an SPSS add-in, was used to generate the matched experimental and control datasets presented in this article.
Data source location	- Institutions: Niger State Polytechnic/Federal Polytechnic/Federal Polytechnic/Nasarawa State Polytechnic - City/Town/Region: - Zungeru, Bida, Nasarawa, Lafia - Country: Nigeria - Latitude and longitude (and GPS coordinates, if possible) for collected samples/data: Zungeru - 9.8097° N, 6.1553° E (GPS: 9.74355, 6.13257)/ Bida - 9.0797° N, 6.0097° E (GPS: 9.03945, 6.00795 / Nasarawa - 8.5475° N, 7.7118° E (GPS: 8.46439, 7.6439)/Lafia (GPS: 8.54550, 8.53598)
Data accessibility	**Repository name**: Mendeley Data and Zenodo. **Data identification number**: Mendeley Data: DOI: 10.17632/43m7g42bcp.2 Zenodo: DOI: 10.5281/zenodo.6641277. **Direct URL to data**: Mendeley Data: https://data.mendeley.com/datasets/43m7g42bcp
	Zenodo: https://zenodo.org/record/6940086#.YuPl2EVKjIU

**Value of the Data**

• These datasets contribute empirical data on the effect of Scratch, a block-based programming language, on students’ achievements in a college first-year programming course (CS1).
• Academic researchers and students looking at how programming pedagogy affects CS1 achievement, as well as computing instructors planning to use Scratch in a college course, can benefit from using these data.
• With these data, a researcher can generate the effect size likely to be detected in an experiment comparing the effects of Scratch and conventional programming languages on the achievements of first-year college students. This input is required by a researcher when performing a power analysis to determine the required sample sizes for the treatment groups.
• They can also be used to test factors that may moderate CS1 achievement, such as previous achievements in English, mathematics, and physics, age or gender.
• These data can be used to reproduce or replicate experiments comparing the effects of constructionist Scratch and conventional pedagogies on CS1 students’ achievements. This can be achieved by employing the same unmatched or matched data, or by using a matching algorithm like the Coarsened Exact Matching (CEM) to generate randomly matched samples from the unmatched data.
• Other research questions or hypotheses can also be tested with these data. For example, the data collected included both the conceptual and algorithmic knowledge that the students provided in their answers to the open-ended questions in the achievement tests. From these, a researcher can explore the knowledge gained by the participants from the use of both pedagogies.

## 1 Data Description

The data presented in this article were obtained from a research project that compared the effects of a constructionist Scratch programming intervention and conventional programming instruction on the achievements of first-year college computer science students. Constructionism, a variant of the constructivist theory, is an educational philosophy propounded by the South African American mathematician and computer scientist Seymour Papert. Defined as a theory of learning and making, constructionism argues that students can engage better with knowledge if teachers provide them with the freedom to express their creative potentials as they construct and share artefacts of interest with their peers [2]. Scratch, the most popular block-based programming language, is a product of the constructionist philosophy. While the constructionist class experienced an inquiry-based learning with the teacher presenting Scratch programming demos and students developing Scratch codes, the conventional instruction had lectures and labs (with students employing Visual Basic, a textual programming language). In this section, we present the demographic and achievement data from both treatment groups as provided in the repository [1].

### 1.1 Demographic Data

These datasets contained demographic information such as gender, age, and educational, programming, and artistic backgrounds. These variables provide a means for operationalising and measuring constructs that are sometimes found to moderate CS1 students’ achievements. Although some variables had values reported by the participants, others represented indices computed from their self-reported data.

### 1.2 Achievement Data

We gathered the achievement data using the open-ended questions from the pretest and posttest. The questions were split into 2 categories: conceptual programming knowledge and computational thinking. By testing for computational thinking, we made the tests language-independent since both treatment groups were exposed to different programming languages. In doing this, we assessed students’ activities that resemble constructing, explaining, and tracing program codes.

We evaluated the students’ answers to the questions in the achievement tests, employing a combined taxonomy (Bloom and SOLO), as used in [3].

The taxonomy used in the rubric for grading the tests had 3 categories, from lowest to highest: unistructural, multistructural and cognitive classes.

Also, each category had 3 cognitive levels, from lowest to highest: understanding, applying, and creating.

Unistructural cognition denotes a student's limited knowledge of a body of concepts and local perspective. The student fails to connect between related ideas and misses the other points or ideas.

When a student responds with multiple ideas or concepts in their answers, this is a sign of multistructural knowledge. However, the student did not connect these related concepts.

The relational cognitive category assumes that the student knows every related idea or concept and can connect them in the correct way.

Therefore, a student demonstrates the highest ability when their answer indicates relational creation and the lowest when their answer shows a unistructural understanding.

In the datasets provided in the repository [1], both demographic and achievement data were combined into SPSS file or Microsoft Excel files. To simplify the presentation in this article, we divide the contents of a file in the repository into four tables (Tables 1–4).

**Table 1:** Data (variables) from CSPROQ

Variable	Description	Code	Level
Gender	Gender (Self-reported)	1 Male	Nominal
		2 Female	

Age	Age Group (Self-reported)	1 16 - 18	Ordinal
		2 19–21	

		3 22–24	
		4 > 24	
		6 Others	

PriorAcademicBackground	Academic Background Index (System-computed)	1 Low	Ordinal
		2 Average	
		3 High	

PriorProgrammingLearning	Prior programming learning index (System-computed)	0 None	Ordinal
		1 Some	

PriorProgramWriting	Prior Program Writing Index (System-computed)	0 None	Ordinal
		1 Some	

PriorVisualArt	Visual Art Background index (System-computed)	0 None	Ordinal
		1 Some	

English	Secondary school O-Level matric English grade (self-reported)	1–9 (highest to lowest)	Ordinal

EnglishGP	English grade point (System-computed)	0–8 (lowest to highest)	Scale

Math	Secondary school O-Level matric Math's grade (Self-reported)	1–9 (highest to lowest)	Ordinal

MathsGP	Math's grade point (System-computed)	0–8 (lowest to highest)	Scale

Physics	Secondary school O-Level matric Physics grade (Self-reported)	1–9 (highest to lowest)	Ordinal

PhysicsGP	Physics’ grade point (System-computed)	0–8 (lowest to highest)	Scale

LearntInPrySchl	Prior Programming learning (Self-reported)	0 No	Nominal
		1 Yes	

LearntInSecSchool	Prior Programming learning (Self-reported)	0 No	Nominal
		1 Yes	

LearntAtITSchl	Prior Programming learning (Self-reported)	0 No	Nominal
		1 Yes	

LearntAtITPark	Prior Programming learning (Self-reported)	0 No	Nominal
		1 Yes	

OnTheInternet	Prior Programming learning (Self-reported)	0 No	Nominal
		1 Yes	

FromTextBook	Prior Programming learning (Self-reported)	0 No	Nominal
		1 Yes	

Others	Prior Programming learning (Self-reported)	0 No	Nominal
		1 Yes	

CCC#	Prior Program writing (Self-reported)	0 No	Nominal
		1 Yes	

HTML	Prior Program writing (Self-reported)	0 No	Nominal
		1 Yes	

Java	Prior Program writing (Self-reported)	0 No	Nominal
		1 Yes	

JavaScript	Prior Program writing (Self-reported)	0 No	Nominal
		1 Yes	

BasicVisualBasic	Prior Program writing (Self-reported)	0 No	Nominal
		1 Yes	

Python	Prior Program writing (Self-reported)	0 No	Nominal
		1 Yes	

MATLAB	Prior Program writing (Self-reported)	0 No	Nominal
		1 Yes	

SQL	Prior Program writing (Self-reported)	0 No	Nominal
		1 Yes	

Scratch	Prior Program writing (Self-reported)	0 No	Nominal
		1 Yes	

Other	Prior Program writing (Self-reported)	0 No	Nominal
		1 Yes	

PlayingComputerGames	Self-reported Likert-scale data	1 Not all	Ordinal
		2 Sometimes	
		3 Often	
		4 Generally	
		5 Almost Always	

DrawingOnTheComputer	Self-reported Likert-scale data	1 Not all	Ordinal
		2 Sometimes	
		3 Often	
		4 Generally	
		5 Almost Always	

BuildingArtWorks	Self-reported Likert-scale data	1 Not all	Ordinal
		2 Sometimes	
		3 Often	
		4 Generally	
		5 Almost Always	

WorkingWithVideos	Self-reported Likert-scale data	1 Not all	Ordinal
		2 Sometimes	
		3 Often	
		4 Generally	
		5 Almost Always	

Table 1 contains data from the CS1 Student Profile Questionnaire (CSPROQ). We performed some pre-processing in Microsoft Excel before moving the data to SPSS. As a result, the table now includes self-reported data as well as data generated from the self-reported data using Excel formulas. For instance, from English, Math, and Physics, respectively, the EnglishGP, MathGP, and PhysicsGP were computed. PriorAcademicBackground is an index that indicates a participant's prior academic performance. EnglishGP, MathsGP, and PhysicsGP were used to compute this index. PriorProgrammingLearning is also an index. It indicated the level of a participant's prior learning of programming. This was calculated based on values in variables LearntInPrimarySchool, LearntInSecSchool, LearntAtITSchl, LearntAtITPark, OnTheInternet, and FromTextBook. PriorProgramWriting is also an index aimed at measuring level of students' prior-to-college experience with writing programs. It was derived from answers to questions about participants' prior programming experience in programming languages like C/C++/C#, HTML, Java, JavaScript, Basic/VisualBasic, Python, MATLAB, SQL, Scratch, and Others. Using four self-reported Likert-scale variables—PlayingComputerGames, DrawingOnTheComputers, BuildingArtworks, and WorkingWithVideos—the PriorVisualArt index was computed to measure the degree of prior visual artistic experience of the participants.

Before taking programming classes in one of the 2 modes, the data that were gathered from the participants are listed in Table 2. The variables in this pretest instrument demand that participants respond to some open-ended questions. The questions consist of 2 categories: conceptual programming knowledge and computational/algorithmic thinking. Variables CMU1 to CMU10 refer to the first category, while the remaining variables refer to the second category. CTOTAL20 represents the total score computed from the values of CMU1 to CMU10. The CQTOTAL represents the total score of the computational/algorithmic thinking questions. The total score for the pretest was 50, as represented by the PretestScore50.

**Table 2:** Data (variables) in the pretest (IPAT_1_).

CMU1	Pretest_Conceptual Multistructural Understanding_Question1	0–2	Scale
CMU2	Pretest_Conceptual Multistructural Understanding_Question2	0–2	Scale
CMU3	Pretest_Conceptual Multistructural Understanding_Question3	0–2	Scale
CMU4	Pretest_Conceptual Multistructural Understanding_Question4	0–2	Scale
CMU5	Pretest_Conceptual Multistructural Understanding_Question5	0–2	Scale
CMU6	Pretest_Conceptual Multistructural Understanding_Question6	0–2	Scale
CMU7	Pretest_Conceptual Multistructural Understanding_Question7	0–2	Scale
CMU8	Pretest_Conceptual Multistructural Understanding_Question8	0–2	Scale
CMU9	Pretest_Conceptual Multistructural Understanding_Question9	0–2	Scale
CMU10	Pretest_Conceptual Multistructural Understanding_Question10	0–2	Scale
CTOTAL20	System-computed score (Out of 20)	0–20	Scale
Q1MA1	Pretest_Question1_Multistructural Applying	0–5	Scale
Q1RU2	Pretest_Question1_Relational Understanding	0–5	Scale
Q1TOTAL10	System-computed Score for Question 1	0–10	Scale
Q2MA1	Pretest_Question2_Multistructural Applying	0–6½	Scale
Q2MC2	Pretest_Question2_Multistructural Creating	0–3½	Scale
Q2TOTAL10	System-computed Score for Question 2	0–10	Scale
Q3UU1	Pretest_Question3_Unistructural Understanding1	0–1	Scale
Q3UU2	Pretest_Question3_Unistructural Understanding2	0–1	Scale
Q3MA3	Pretest_Question3_Multistructural Applying3	0–1½	Scale
Q3MA4	Pretest_Question3_Multistructural Applying4	0–1½	Scale
Q3MA5	Pretest_Question3_Multistructural Applying5	0–1½	Scale
Q3RA6	Pretest_Question3_Relational Applying6	0–2	Scale
Q3RA7	Pretest_Question3_Relational Applying6	0–1½	Scale
Q3TOTAL10	System-computed Score for Question 3	0–10	Scale
CQTOTAL30	System-computed Score for Q1, Q2, and Q3	0–30	Scale
PretestScore50	System-computed	0–50	Scale

Table 3 presents the data collected from participants after exposing them to programming in the 2 classes. Although with some reordering of questions, this posttest contains the same variables as in the pretest (Table 2).

**Table 3:** Data (variables) in the posttest (IPAT_2_).

CMU1_A	Posttest_Conceptual Multistructural Understanding_Question1	0–2	Scale
CMU2_A	Posttest_Conceptual Multistructural Understanding_Question2	0–2	Scale
CMU3_A	Posttest_Conceptual Multistructural Understanding_Question3	0–2	Scale
CMU4_A	Posttest_Conceptual Multistructural Understanding_Question4	0–2	Scale
CMU5_A	Posttest_Conceptual Multistructural Understanding_Question5	0–2	Scale
CMU6_A	Posttest_Conceptual Multistructural Understanding_Question6	0–2	Scale
CMU7_A	Posttest_Conceptual Multistructural Understanding_Question7	0–2	Scale
CMU8_A	Posttest_Conceptual Multistructural Understanding_Question8	0–2	Scale
CMU9_A	Posttest_Conceptual Multistructural Understanding_Question9	0–2	Scale
CMU10_A	Posttest_Conceptual Multistructural Understanding_Question10	0–2	Scale
CTOTAL20_A	Posttest System-computed score (/20)	0–20	Scale
Q2MA1_A	Posttest_Question1_Multistructural Applying	0–5	Scale
Q2MC2_A	Posttest_Question2_Multistructural Creating2	0–5	Scale
Q2TOTAL10_A	Posttest System-computed Score for Question 2	0–10	Scale
Q3UU1_A	Posttest_Question3_Unistructural Understanding1	0–1	Scale
Q3UU2_A	Posttest_Question3_Unistructural Understanding2	0–1	Scale
Q3MA3_A	Posttest_Question3_Multistructural Applying3	0–1½	Scale
Q3MA4_A	Posttest_Question3_Multistructural Applying4	0–1½	Scale
Q3MA5_A	Posttest_Question3_Multistructural Applying5	0–1½	Scale
Q3RA6_A	Posttest_Question3_Relational Applying6	0–2	Scale
Q3RA7_A	Posttest_Question3_Relational Applying6	0–1½	Scale
Q3TOTAL10_A	Posttest_System-computed Score for Question 3	0–1½	Scale
Q1MA1_A	Pretest_Question1_Multistructural Applying1	0–5	Scale
Q1RU2_A	Posttest_Question1_Relational Understanding2	0–5	Scale
Q1TOTAL10_A	System-computed Score for Question 1	0–10	Scale
CQTOTAL30_A	System-computed Score for Q1, Q2, and Q3	0–30	Scale
PosttestScore50	System-computed	0–50	Scale

The variables obtained by rounding the pre-test, post-test, and gain scores (the difference between the pre-test and post-test) to 100 are shown in Table 4.

**Table 4:** Conversion of scores to 100.

Pretest	Pretest (System-computed)	(0–100)	Scale
Posttest	Posttest (System-computed)	(0–100)	Scale
GainScore	GainScore (System-computed)	(0–100)	Scale

Two CS1 cohorts from 2 polytechnics are represented in Table 5, showing the descriptive summaries of the data collected from them. Participants who enrolled and were instructed in the 2 programming learning modes make up both samples. The minimum entry age to university in Nigeria is 16. This informed our use of participants from that age in our data collection. However, this raises some ethical questions as regards consent. We provide answers to that in the ethical statements section of this article.

**Table 5:** Demography and achievements of intact classes (dataset1)

		Primary Independent (Treatment) Variable							
		Constructionist Class				Conventional Class			
			Dependent Variables						
Secondary Independent Variables		N	(%)	Mean IPAT Pretest	Mean IPAT Posttest	N	(%)	Mean IPAT Pretest	Mean IPAT Posttest
Gender	Male	80	(61.0)	25.82	47.88	78	(65.0)	22.42	37.79
	Female	36	(39.0)	22.44	50.17	42	(35.0)	18.93	36.57
	Total	116				120			

Age	16–18	16	(13.8)	31.13	51.25	10	(8.3)	24.40	43.00
	19–21	64	(55.2)	26.09	48.66	57	(47.5)	21.86	37.74
	22–24	27	(23.3)	19.70	48.96	40	(33.3)	19.60	36.85
	> 24	9	(7.7)	19.33	42.22	12	(10.0)	21.67	38.67
	Others	-	-	-	-	1	(0.8)	10.00	20.00
	Total	116				120			

Prior Academic Level	Low	79	(68.1)	25.95	48.96	81	(67.5)	19.22	36.79
	Average	35	(30.2)	22.29	47.60	33	(27.5)	26.45	40.61
	High	2	(1.7)	22.00	51.00	6	(5.0)	19.00	27.33
	Total	116				120			

Prior Program Writing	None	111	(95.7)	24.22	48.52	108	(90.0)	20.56	36.89
	Some	5	(4.3)	37.20	50.00	12	(10.0)	27.00	41.67
	Total	116				120			

Prior Visual Art	None	27	(23.3)	20.59	40.89	40	(33.3)	19.95	36.60
	Some	89	(76.7)	26.04	50.92	80	(66.7)	21.83	37.75
	Total	116				120			

Table 6 displays the descriptive summaries of the matched samples generated by CEM from dataset1 (Table 5). The samples consist of cases chosen at random from each treatment group in Table 5. They were then assigned to the corresponding treatment groups in dataset2 (Table 6). Matching was to ensure that we have equivalents samples in the 2 treatment groups in dataset2. Pretest scores, gender, age, prior academic level, prior program writing and prior visual artistic abilities of students in the intact classes are among the covariates used to match samples.

**Table 6:** Demography and achievements of matched samples (dataset2)

		Primary Independent (Treatment) Variables							
		Constructionist Class				Conventional Class			
			Dependent Variables						
Secondary Independent Variables		N	(%)	Mean IPAT Pretest	Mean IPAT Posttest	N	(%)	Mean IPAT Pretest	Mean IPAT Posttest
Gender	Male	28	(68.3)	22.21	48.71	28	(68.3)	22.29	38.86
	Female	13	(31.7)	20.77	49.23	13	(31.7)	21.54	41.23
	Total	41				41			

Age	16–18	4	(9.8)	27.50	54.50	4	(9.8)	27.50	51.50
	19–21	24	(58.5)	21.17	46.83	24	(58.5)	22.00	36.58
	22–24	12	(29.3)	20.50	52.17	12	(29.3)	20.00	42.83
	> 24	1	(2.4)	28.00	36.00	1	(2.4)	26.00	26.00
	Total	41				41			

Prior Academic Level	Low	32	(78.0)	21.81	48.00	32	(78.0)	21.75	39.69
	Average	9	(22.0)	21.56	52.00	9	(22.0)	23.11	39.33
	High	-		-	-				
	Total	41				41			

Prior Program Writing	None	41	(100.0)	21.76	48.88	41	(100.0)	22.05	39.61
	Some		(0.0)	-	-		(0.0)	-	-
	Total	41				41			

Prior Visual Art	None	7	(17.1)	21.71	40.86	7	(17.1)	21.14	41.14
	Some	34	(82.9)	21.765	50.53	34	(82.9)	22.23	39.29
	Total	41				41			

Descriptive summaries of the demographic and achievement data from 2 new cohorts of CS1 students who participated in the following session are presented in Table 7.

**Table 7:** Demography and achievements of intact classes (dataset3)

		Primary Independent (Treatment) Variable							
		Constructionist Class				Conventional Class			
			Dependent Variables						
Secondary Independent Variables		N	(%)	Mean IPAT Pretest	Mean IPAT Posttest	N	(%)	Mean IPAT Pretest	Mean IPAT Posttest
Gender	Male	76	(79.2)	13.13	28.53	59	(68.6)	19.29	26.81
	Female	20	(20.8)	14.60	23.90	27	(31.4)	17.26	29.41
	Total	96				86			

Age	16–18	12	(12.5)	16.83	28.83	15	(17.4)	23.07	31.07
	19–21	44	(45.8)	13.32	27.50	38	(44.2)	19.18	27.68
	22–24	34	(35.4)	12.53	27.24	28	(32.6)	14.43	25.71
	> 24	5	(5.2)	14.80	29.00	5	(5.8)	25.00	27.60
	Others	1	(1.0)	2.00	19.00	-	-	-	-
	Total	96				86			

Prior Academic Level	Low	80	(83.3)	12.98	27.35	53	(61.6)	18.38	27.13
	Average	14	(14.6)	17.57	29.43	33	(38.4)	19.09	28.42
	High	2	(2.1)	3.00	23.00	-	-	-	-
	Total	96				86			

Prior Program Writing	None	86	(89.6)	13.21	27.67	74	(86.0)	18.36	26.92
	Some	10	(10.4)	15.40	26.60	12	(14.0)	20.42	32.00
	Total	96				86			

Prior Visual Art	None	36	(37.5)	10.17	24.50	44	(51.2)	17.82	28.05
	Some	60	(62.5)	15.40	29.40	42	(48.8)	19.52	27.19
	Total	96				86			

Table 8 gives the summaries of the matched samples (dataset4) obtained by using CEM to match cases from the 2 treatment groups in Table 7. Samples were matched on pretest scores, gender, age, prior academic level, prior program writing and prior visual artistic abilities of students in the intact classes (dataset3).

**Table 8:** Demography and achievement of matched samples (dataset4).

		Primary Independent (Treatment) Variable							
		Constructionist Class				Conventional Class			
		Dependent Variables							
Secondary Independent Variables		N	(%)	Mean IPAT Pretest	Mean IPAT Posttest	N	(%)	Mean IPAT Pretest	Mean IPAT Posttest
Gender	Male	33	(78.6)	13.30	29.42	27	(64.3)	16.56	22.81
	Female Total	9 42	(21.4)	16.00	24.78	15 42	(35.7)	10.20	24.53

Age	16–18	6	(14.3)	14.33	30.00	6	(14.3)	12.50	25.67
	19–21	17	(40.5)	13.88	29.35	17	(40.5)	15.41	24.00
	22–24	17	(40.5)	13.06	25.50	16	(38.1)	12.53	21.41
	>24 Total	2 42	(4.8)	17.33	35.67	3 42	(7.1)	25.00	29.00

Prior Academic Level	Low	32	(76.2)	13.03	28.25	32	(76.2)	13.63	23.06
	Average	10	(23.8)	16.60	29.00	10	(23.8)	16.40	24.60
	High Total	- 42		-	-	- 42		-	-

Prior Program Writing	None	41	(97.6)	13.44	28.02	41	(97.6)	13.85	22.98
	Some Total	1 42	(2.4)	32.00	45.00	1 42	(2.4)	32.00	42.00

Prior Visual Art	None	22	(52.4)	11.18	27.09	22	(52.4)	11.64	23.73
	Some Total	20 42	(47.6)	16.85	29.90	20 42	(47.6)	17.20	23.10

## 2 Experimental Design, Materials and Methods

### 2.1 Research Design

**Fig. 1:**
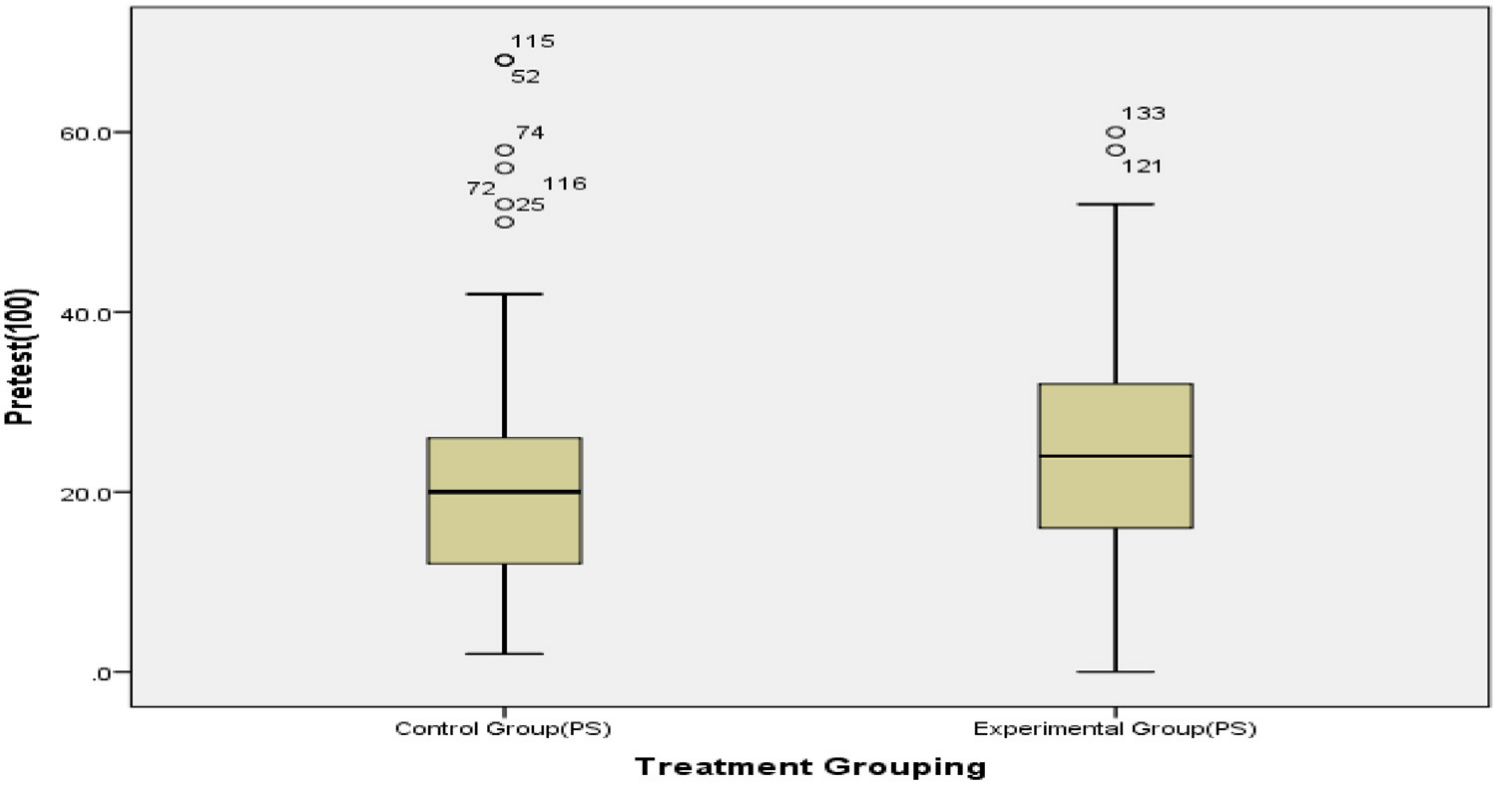
Box plot indicating outliers in the dataset before matching.

**Fig. 2:**
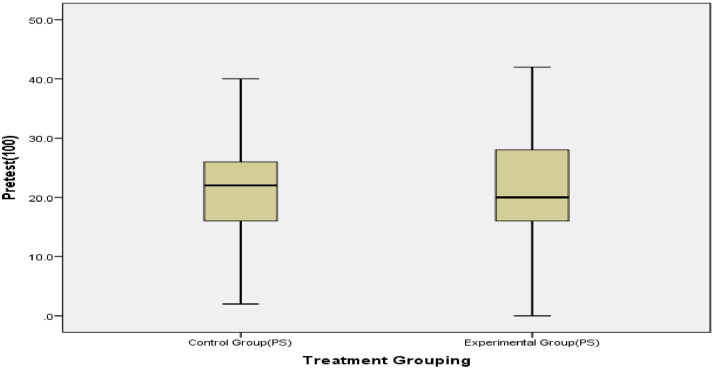
Box plot indicating no outlier after matching.

We employed a quasi-experimental, non-equivalent pre-test–post-test control group design. With the weakness arising from this inability to assign participants randomly to treatment classes, the research design was strengthened by pretesting and using the Coarsened Exact Matching (CEM) algorithm to generate matched treatment groups (Fig. 6). Another advantage of employing CEM is that it removed outliers from the unmatched data, generating equivalent samples for data analysis (See Fig. 1, Fig. 2). Interested users can download CEM freely as an SPSS add-in from https://projects.iq.harvard.edu/cem-spss/pages/installation. Following the installation, CEM will be found in the Analyze menu in the SPSS program.

### 2.2 Setting

**Fig. 3:**
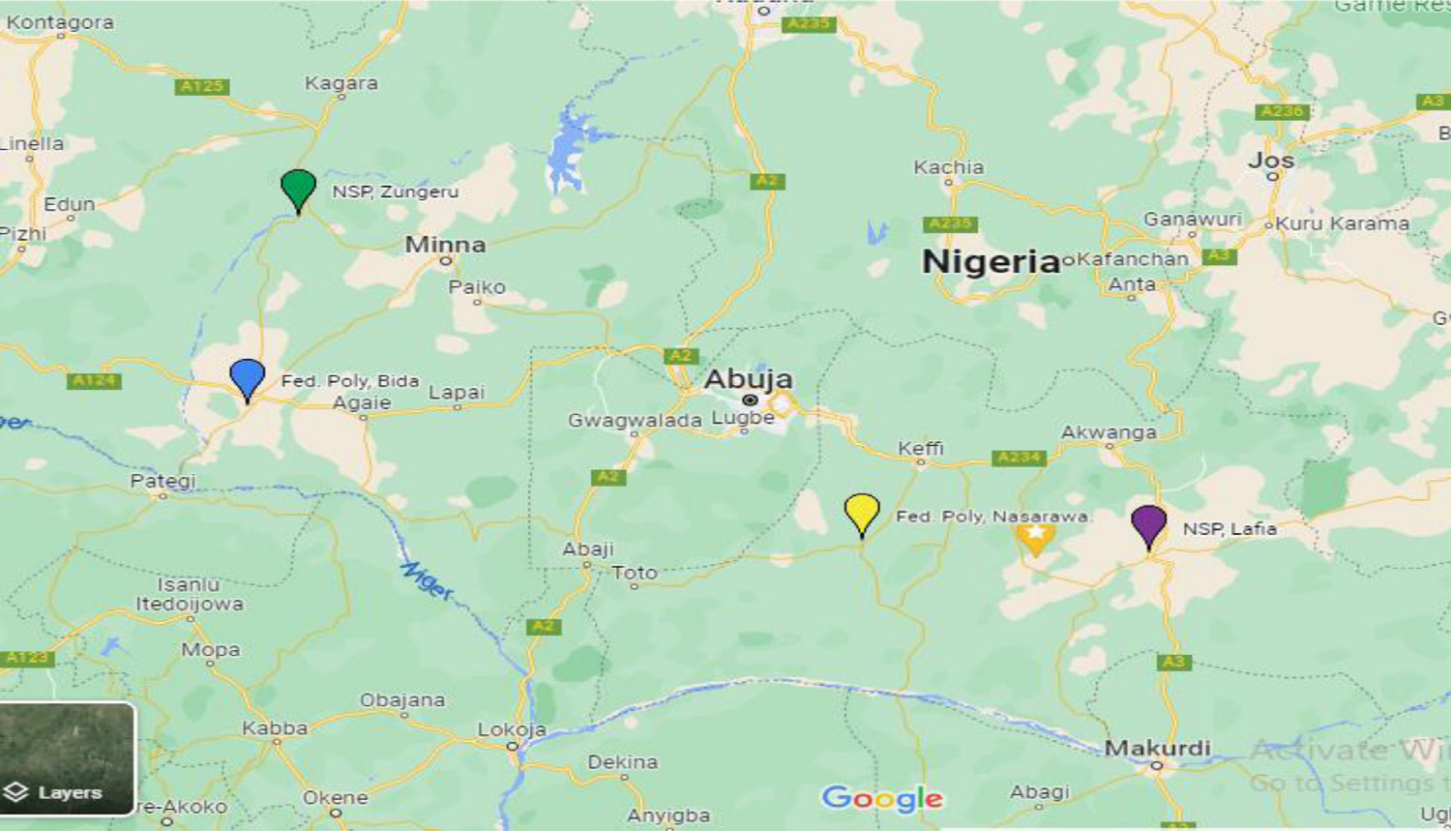
The four data collection sites - selected polytechnics in central Nigeria.

Data collection took place in four selected public polytechnics in 2 states of north-central Nigeria (Fig. 3). Niger State Polytechnic Zungeru (NSPZ) has its main campus in Zungeru, a rural town and former capital of the colonial northern protectorate of Nigeria. The NSPZ admits mainly Niger state indigenes, with most inhabitants working as agrarians, artisans, traders, and civil servants. Federal Polytechnic Bida (FPB) is in Bida, the second largest town in Niger state. Being a federal institution, the FPB admits a large population of students from neighbouring southwestern and northern central states. Another institution, the Federal Polytechnic Nasarawa (FPN), is in Nasarawa State. FPN is in a rural town, but like FPB and with its proximity to Abuja, Nigeria's capital, it enrols large student population from various parts of Nigeria. The fourth site is another state-owned institution, the Nasarawa State Polytechnic Lafia (NSPL), now renamed Isa Mustapha Agwai 1 Polytechnic, located in Lafia, the state capital.

The first experiment was conducted with FPB and FPN representing the control and experimental sites respectively, during the 2014/2015 session. The second experiment was conducted during the 2015/2016 session with new cohorts of students in the NSPZ, FPN, and NSPL. NSPZ represented the experimental group, whereas the other sites were the control groups. However, the datasets presented in this article did not included data from the NSPL.

### 2.3 Sampling

**Fig. 4:**
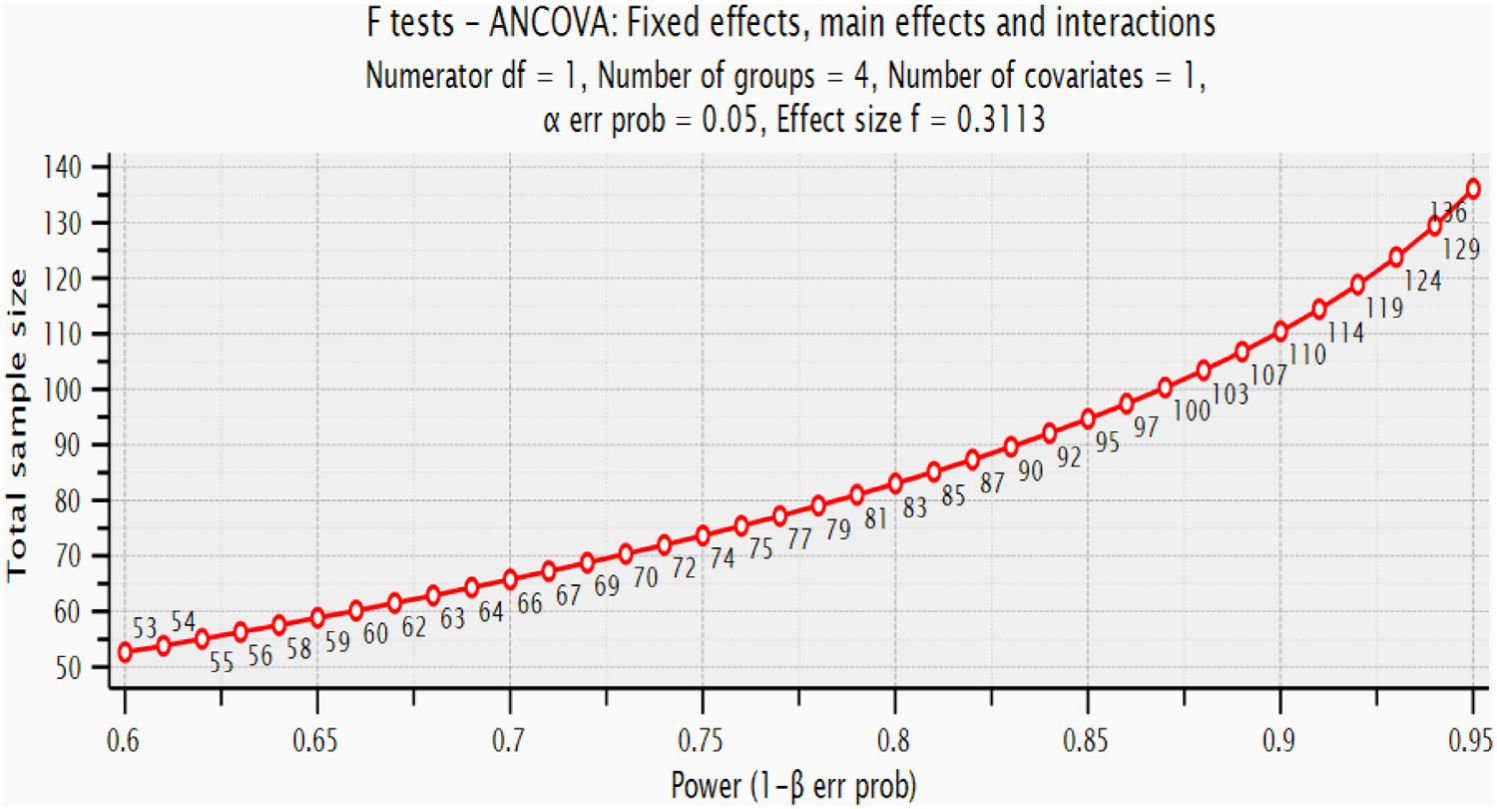
Power analysis to determine the main study sample size.

The same sampling procedure was followed to collect data during the 2 experiments. A purposive sampling technique was employed to select institutions that were randomly assigned to treatment groups. Using CEM, we generated from dataset1 matched samples (*n* = 82, with randomly assigned 41 cases in each treatment group). This resulted in dataset2 shown in Table 9. We conducted an ANCOVA of the dataset2 using SPSS version 23. This provided one input (i.e., effect size) required for the power analysis. We obtained a partial eta-squared value of 0.094, indicating a moderate effect. This value agrees with the value obtained from a meta-analysis comparing the effects of block-based and textual programming languages on student achievements [4]. G*Power version 3.1.9.2 software was used to determine the sample size for dataset3. As Fig. 4 suggests, to detect an effect from the treatment at a power of 0.8, a *p*-value of 0.05, and a moderate effect size of *f* = 0.3113, we would require a sample of 83. With this input, using CEM, we generated from the dataset3, a matched sample (*n* = 84) shown in Table 9.

**Table 9:** Sampling frames.

	Constructionist Scratch Class(Experimental Group)			Conventional CS1 Class(Control Group)			
Sample	Male	Female	Total	Male	Female	Total	Sample Total
**dataset1**	80	36	116	78	42	120	236
**dataset2**	28	13	41	28	13	41	82
**dataset3**	76	20	96	59	27	86	182
**dataset4**	27	15	42	27	15	42	84

### 2.4 Instruments

**Fig. 5:**
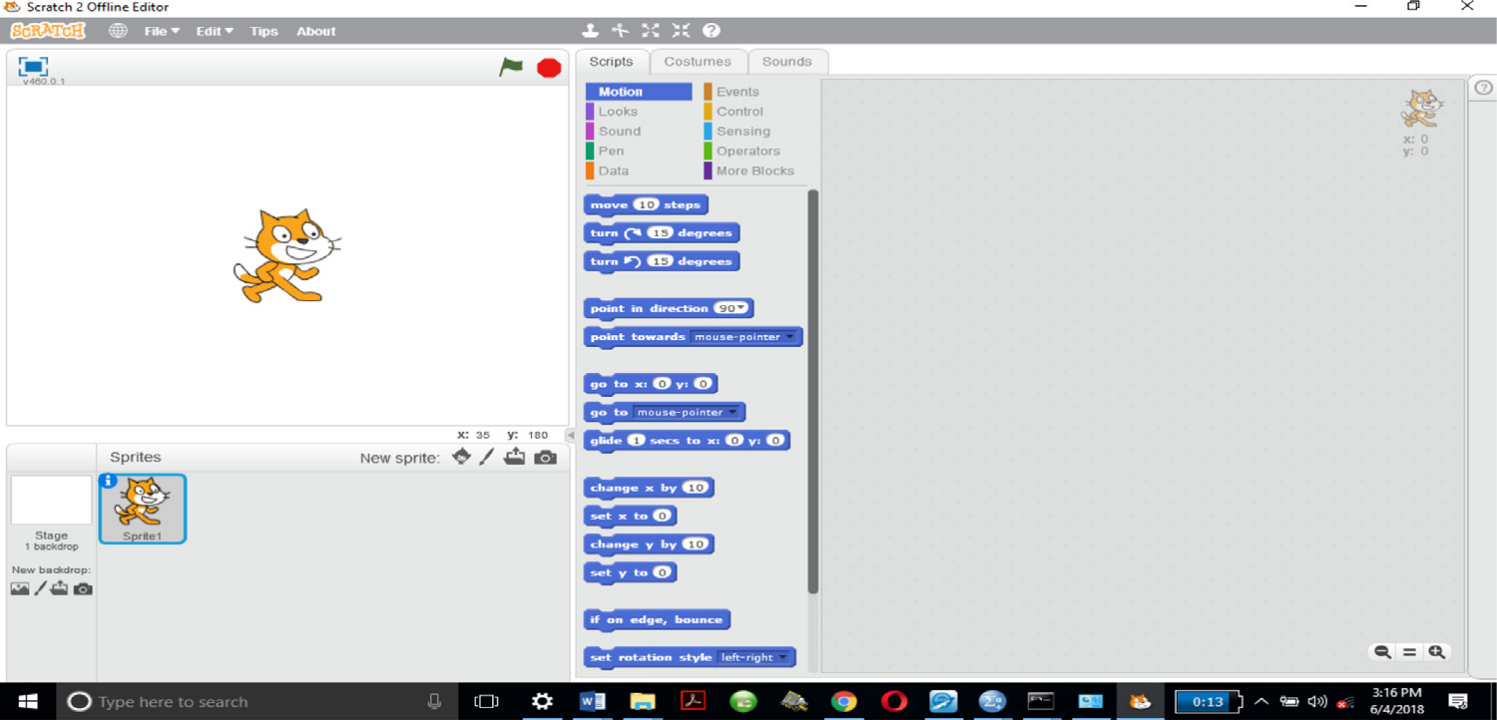
Scratch 2.0 programming environment.

We employed Scratch 2.0 environment (Fig. 5) in the experimental class. Developed by the Lifelong Kindergarten Group at the MIT Media Lab USA, Scratch (current version 3.0) is freely available at https://scratch.mit.edu/download.

Adapting from prior research [3], we developed 2 instruments: the CS1 Student Profile Questionnaire (CSPROQ) and Introductory Programming Achievement Test (IPAT). The participants provided demographic data with the CSPROQ and achievement data using the IPAT. IPAT was used as a pretest, then with reordering questions, as a posttest. An author in [3] and two researchers validated both the CSPROQ and IPAT. Table 10 presents information on CSPROQ and IPAT's reliability. Details of other data validation tests conducted are provided in the repository [1]

**Table 10:** Reliability analyses – CSPROQ and IPAT.

Construct	Items	Ordinal Alpha
CSPROQ:		
Academic background	3	0.72
Programming background	17	0.85
Visual art background	5	0.75
IPAT:		
Programming concepts/Computational thinking	27	0.84*

NB. *Cronbach Alpha.

### 2.5 Data Collection Procedure

**Fig. 6:**
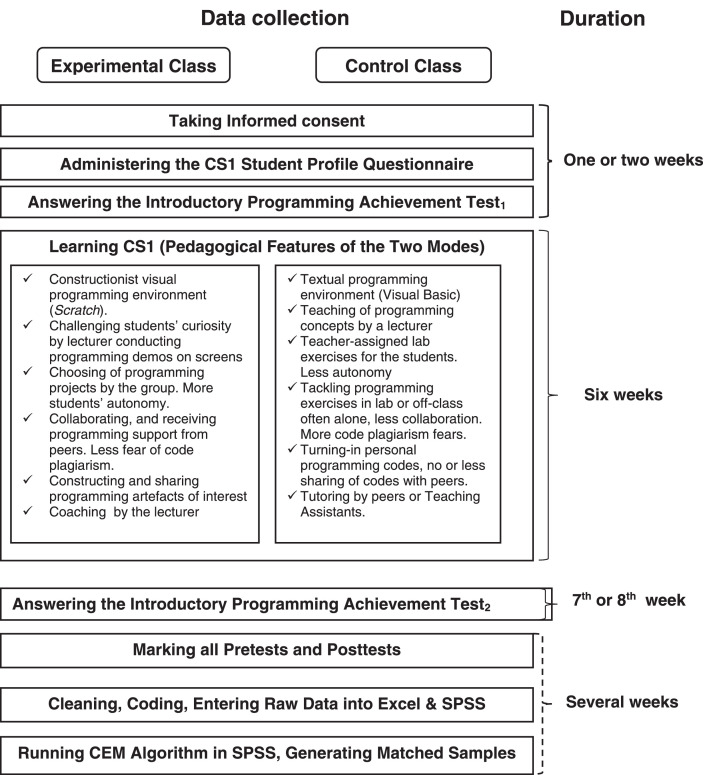
Data collection procedure.

These experimental data were acquired from a research project that spanned 2 academic sessions: 2014/2015 and 2015/2016. As shown in Fig. 6, each experiment started by administering CS1 students' profile questionnaires to the participants. Before programming instructions began, participants in both groups took the introductory programming achievement test (IPAT_1_) as a pretest. The first author taught both classes in two-hour weekly sessions for six weeks. Fig. 6 highlights the activities and features of both instruction modes. Then, subjects in both groups took the posttest, that is, IPAT_2_ which contained the same questions as IPAT_1_, but with some reordering.

### 2.6 Data Validation

To use these data to answers specific research questions or test hypotheses, they need to satisfy some assumptions for required statistical tests. We provide additional documentation in the repository [1] detailing the specific tests that were conducted to validate the data.

## Ethics Statements

Before commencing the research project, the Institute for Science and Technology Education-Sub Research Ethics Review, and College of Science, Engineering, and Technology Research and Ethics committees of the University of South Africa scrutinised and granted ethical approvals (No. 2015_CGS/ISTE_016) for the collection of these data. Then, we requested and obtained the approvals from the managements of participating polytechnics. Lastly, the research participants, after duly informing them about the nature of the project, signed the informed consent forms. Few participants between the ages of 16 and 17 took part in the study, raising some ethical questions since we did not obtain their parental approvals. However, the nature of the research and context provide some answers. The research is a low risk involving intact first-year computer science classes with one group learning to program in the conventional way and the other group learning in a constructivist inquiry-based pedagogy, during a six-week period.

In Nigeria, as in Sweden [5], minors between the ages of 16 and 17 can participate in research  without obtaining parental approval, as long they have the capacity to give their informed consents. Nevertheless, as stated earlier, data collection took place only after we have obtained approvals of participating institutions. We have provided copies of the ethical clearance certificates, participating institutions’ approvals, and the informed consent form in the supplementary files.

## CRediT authorship contribution statement

**Oladele O. Campbell:** Conceptualization, Methodology, Data curation, Writing – original draft. **Harrison I. Atagana:** Supervision, Writing – review & editing.

## Declaration of Competing Interest

The authors declare that they have no known competing financial interests or personal relationships that could have appeared to influence the work reported in this paper.

## Data Availability

The Conventional versus Constructionist-Scratch programming instructions and students achievements in higher education CS1 classes (Original data) (Mendeley Data).

## Appendix Supplementary materials

Image, application 1

Image, application 2

Image, application 2
