# Supplementary material for: In search of experimental evidence on Scratch programming and students’ achievements in the first-year college computing class? Consider these datasets
Source: Data Brief. 2022 Sep 24;45:108635. doi: 10.1016/j.dib.2022.108635 (PMC9679459; doi:10.1016/j.dib.2022.108635)
Supplement: Supplementary file 1 [file mmc1.pdf]

CS1 Students Profile Questionnaire (CSPROQ)

A - STUDENTS' DEMOGRAPHIC QUESTIONNAIRE

The purpose of this questionnaire is to provide information showing the backgrounds of first-year computer science students taking part in the above PhD research study. The study is aimed at improving the students' performance in introductory programming course (COM 113). I want to assure you that information you provide shall be kept confidential. So, please feel free to supply information as honest and accurate as possible. Thank you for your willingness to participate in the study.

INSTRUCTION:

Kindly complete all items/questions by **writing** your answer in the (shaded) space provided or you may **tick** [√] or **mark (X**) as appropriate in the space provided.

Name of Polytechnic:________________________________

Identification Number:

1. Gender:

Male	
Female	

2. Age

16 -18 years	

19 -21 years	
22 - 24 years	
Above 24 years	

3. Your 'O' Level grades in the (3) compulsory subjects with which you were admitted into National Diploma one (Indicate the source of each result i.e., WAEC, NECO, NABTEB)

**Subject**	**Grade**	**Examination Body (i.e., WAEC, NECO, etc)**
English		
Mathematics		
Physics		

4. Unified Tertiary Matriculation Exam (UTME) Score Achieved

300-400	
250-299	
200-249	
150-199	

Other (Specify)_______________	

5. Have you learnt programming before?

Yes	
No	

6. If you answered Yes to question 5, where did you learn programming?

Primary School	
Secondary School	
Private IT school	
IT park	
On the Internet	
From teach-yourself programming textbook(s)	
Other (Please specify) ____________	

7. Have you written any computer program before?

Yes	
No	

8. If you answered Yes to question 7, which of these programming language(s) have you written program in before?

C/C++/C#	

HTML	
Java	
JavaScript	
Basic/Visual Basic	
Python	
Matlab	
SQL	
Scratch	
Other (Please specify) _____________	

C.**What level of experience do you have with each of the following items (Scale: Highest is 5, lowest is 1)**

**Response Code** / **Items**	**Almost Always (5)**	**Generally (4)**	**Often (3)**	**Sometimes (2)**	**Not all (1)**
Playing computer games					
Drawing on the computer					

Building or making art works					
Taking or editing videos					

Thank you for your kind participation in this study!

IMPACT OF *SCRATCH* ON THE ACHIEVEMENTS OF FIRST-YEAR COMPUTER SCIENCE STUDENTS IN SOME NIGERIAN POLYTECHNICS

By

Oladele Campbell
Institute for Science and Technology Education
University of South Africa (UNISA), Pretoria. South Africa.

INTRODUCTORY PROGRAMMING ACHIEVEMENT TEST (IPAT)

PRETEST

The purpose of these pretest questions is to measure your current knowledge or ideas about programming. This test is not counted as part of your continuous assessments for the semester. So, feel free and be yourself as you answer the questions. You do not need to copy your colleagues' answers. Your answer(s) provide data in a study aimed at understanding students' problems with COM 113 (Introduction to Programming) and exploring an alternative approach to teaching the course to improve students' performance. So please be sincere and serious in providing answers where you can. I want to assure you that the information you provide shall be kept confidential and used only for research purpose. Thanks for your willingness to participate in this study.

INSTRUCTION:

Kindly complete all items/questions (where you can) by **writing** your answer or **mark (X**) if you do not have an idea about an item in the space provided.

Name of Polytechnic:____

Identification Number:

Concepts

Here is a list of programming concepts. Please kindly write a short explanation of each one. You are expected to write two or more sentences showing your understanding of each concept. Please write clearly and neatly so that the researcher can understand your answer. If a concept is not familiar, write an “X” in the space provided indicating you do not have an idea.

**Concept**	**Explanation (2 marks each)**	For office use Only
Program		CMU1
Algorithm		CMU2
Assignment		CMU3

Output		CMU4
Variable		CMU5
Input		CMU6
Looping structure		CMU7
Selection structure		CMU8

Sequence structure		CMU9
Arithmetic operators		CMU10

Question 1

There are three playing cards laid out in a row on a table; each card is labelled with a number. You are given the following sequence of instructions:

1. compare the number on the left-hand card with the number on the center card

2. if the number on the left-hand card is greater than the number on the center card

   2.1 exchange the two cards

3. compare the number on the center card with the number on the right-hand card

4. if the number on the center card is greater than the number on the right-hand card

   4.1 exchange the two cards

24	2	15

On the table are the following cards:

(a)What will be the numbers on the cards after you carry out the above instructions? **(5 marks)**

Q1MA1

(b) What is the purpose of the above sequence of instructions? **(5 marks)**

Q1RU2

Question 2

Here is a sequence of instructions:

1. Stand at the origin
2. Turn left
3. Carry out step 3.1 10 times:
    3.1 Move 5 steps
4. Turn right
5. Carry out step 5.1 10 times:
    5.1 Move 5 steps
6. Turn right
7. Carry out step 7.1 10 times:
    7.1 Move 5 steps

(a) If you carry out these instructions, you will follow a path that is the form of some letter in English.
Q2MA2

What is it? (You can also draw the path here.) **(1+.5+1+.5+1+.5+1=5.5) marks for drawing the path. 1 mark for identifying the letter)**

(b) Add more instructions at the end of the list of instructions above so that the path obtained will be a square**.** **(3.5 marks)**
Q2MC1

____

____

____

Question 3

Two groups of kids are competing in a relay race. There are 10 kids in each group but here you are given the names of first three kids in each group. Each one runs to the other side of the yard and back, and hands over the baton to the next kid. It takes 5 minutes for each kid in the first group to run back and forth, and 7 minutes for each kid in the second group to run back and forth. The first group consists of Uche followed by Musa followed by Dayo followed by . . . . . The second group consists of Shade followed by Amina followed by Ada followed by . . . . . See the following diagram:

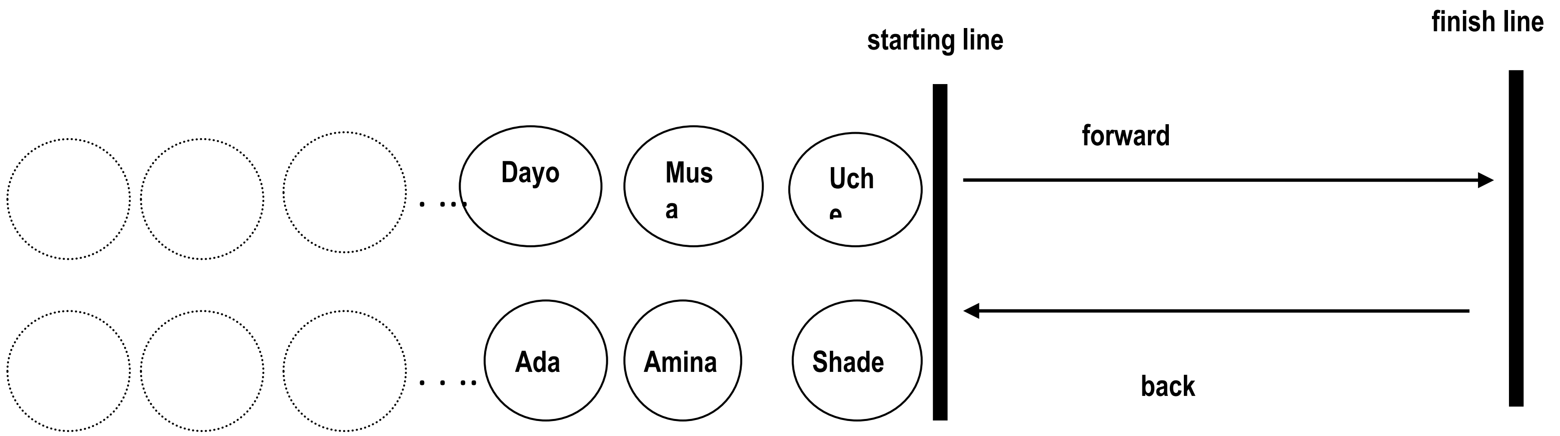

.

(a) Whose turn is it to run after Musa's?____________________________ **(1 marks)**

Q3UU1

(b) Amina's turn comes before whose turn?_________________________ **(1marks)**

Q3UU2

(c) How much time will pass until it is Dayo's turn? Explain briefly_____________________ **(1.5 marks)**

Q3MA3

(d) How much time will pass until all members of the first team finish? Explain briefly _________________ **(1.5 marks)**

Q3MA4

(e) How much time will pass until all members of the second team finish? Explain briefly ___________________ **(1.5 marks)**

Q3MA5

(f) How much time will the whole race take? Explain briefly ____________________________________ **(2 marks)**

Q3RA1

(g) What will happen if Musa loses the baton while he is running? **(1.5marks)**

Q3RA2

____

____

*** This work, IPAT, is an adaptation of a pretest instrument employed in a study "Learning Computer Science Concepts in *Scratch*" by Orni Meerbaum-Salant, Michal Armoni and Moti Ben-Ari, available at** http://stwww.weizmann.ac.il/g-cs/*Scratch*/tests-cs-concepts-in-*Scratch*.zip **used under CC BY.**.

THE IMPACT OF *SCRATCH* ON THE ACHIEVEMENTS OF FIRST-YEAR COMPUTER SCIENCE STUDENTS IN SOME NIGERIAN POLYTECHNICS

By

Oladele Campbell
Institute for Science and Technology Education
University of South Africa (UNISA), Pretoria. South Africa.

INTRODUCTORY PROGRAMMING ACHIEVEMENT TEST (IPAT)

POSTTEST

The purpose of these posttest questions is to measure your current knowledge or ideas about programming. This test is not counted as part of your continuous assessments for the semester. So feel free and be yourself as you answer the questions. You do not need to copy your colleagues' answers. Your answer(s) provide data in a study aimed at understanding students' problems with COM 113 (Introduction to Programming) and exploring an alternative approach to teaching the course so as to improve students' performance. So please be sincere and serious as you provide answers where you can. I want to assure you that the information you provide shall be kept confidential and used only for research purpose. Thanks for your willingness to participate in this study.

INSTRUCTION:

Kindly complete all items/questions (where you can) by **writing** your answer or **mark (X**) if you do not have an idea about an item in the space provided.

Name of Polytechnic:____

Identification Number:____

Concepts

Here is a list of programming concepts. Please kindly write a short explanation of each one. You are expected to write two or more sentences showing your understanding of each concept. Please write clearly and neatly so that the researcher can understand your answer. If a concept is not familiar, write an “X” in the space provided indicating you do not have an idea.

**Concept**	**Explanation (2 marks each)**	For office use Only
Program		CMU1
Algorithm		CMU2
Assignment		CMU3

Output		CMU4
Variable		CMU5
Input		CMU6
Looping structure		CMU7
		CMU8

Selection structure		
Sequence structure		CMU9
Arithmetic operators		CMU10

Question 1

Two groups of kids are competing in a relay race. There are 10 kids in each group but here you are given the names of first three kids in each group. Each one runs to the other side of the yard and back, and hands over the baton to the next kid. It takes 5 minutes for each kid in the first group to run back and forth, and 7 minutes for each kid in the second group to run back and forth. The first group consists of Uche followed by Musa followed by Dayo followed by . . . . . The second group consists of Shade followed by Amina followed by Ada followed by . . . . . See the following diagram:

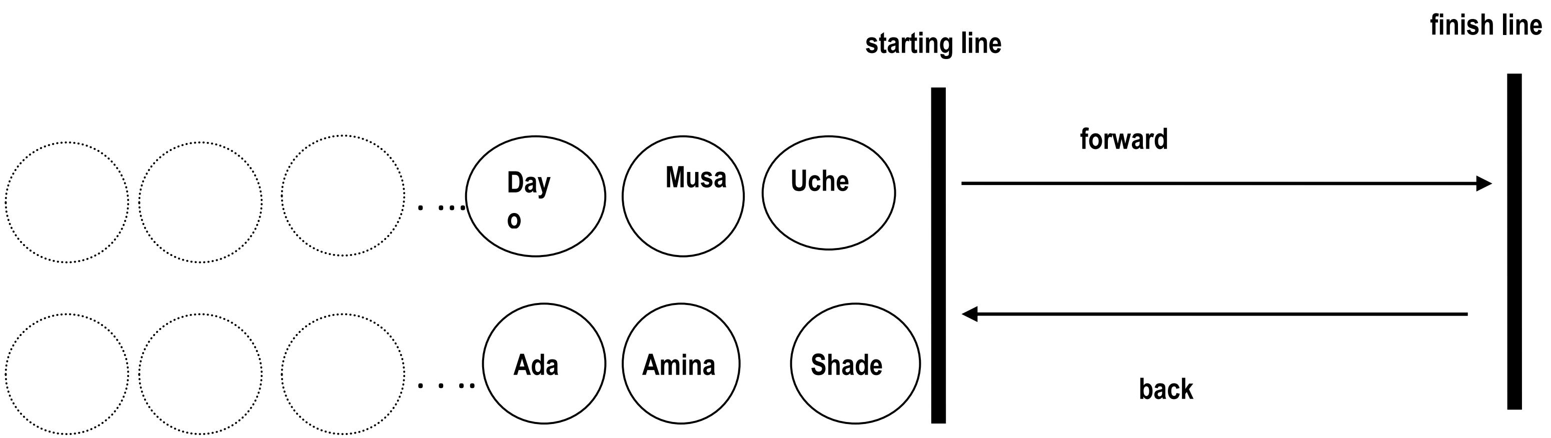

.

(a) Whose turn is it to run after Musa's?____________________________ **(1 marks)**

Q3UU1

(b) Amina's turn comes before whose turn?_________________________ **(1marks)**

Q3UU2

(c) How much time will pass until it is Dayo's turn?______________________ **(1.5 marks)**

Q3MA3

(d) How much time will pass until all members of the first team finish? __________________ **(1.5 marks)**

Q3MA4

(e) How much time will pass until all members of the second team finish?____________________ **(1.5 marks)**

Q3MA5

(f) How much time will the whole race take?______________________________________ **(2 marks)**

Q3RA6

(g) What will happen if Musa loses the baton while he is running? **(1.5marks)**

Q3RA7

____

____

____

____

Question 2

Here is a sequence of instructions:

1. Stand at the origin
2. Turn left
3. Carry out step 3.1 10 times:
   - 3.1 Move 5 steps
4. Turn right
5. Carry out step 5.1 10 times:
   - 5.1 Move 5 steps
6. Turn right
7. Carry out step 7.1 10 times:
   - 7.1 Move 5 steps

(a) If you carry out these instructions, you will follow a path that is the form of some letter in English.
Q2MA1

What is it? (You can also draw the path here.) **(1+.5+1+.5+1+.5+1=5.5) marks for drawing the path. 1 mark for identifying the letter)**

(b) Add more instructions at the end of the list of instructions above so that the path obtained will be a square. **(3.5 marks)**
Q2MC2

____

____

____

Question 3

There are three playing cards laid out in a row on a table; each card is labelled with a number. You are given the following sequence of instructions:

1. compare the number on the left-hand card with the number on the center card
2. if the number on the left-hand card is greater than the number on the center card
   2.1 exchange the two cards
3. compare the number on the center card with the number on the right-hand card
4. if the number on the center card is greater than the number on the right-hand card
   4.1 exchange the two cards

On the table are the following cards: | 8 | | 2 | | 6 |

(a)What will be the numbers on the cards after you carry out the above instructions? | | | | | | **(5 marks)**

Q1MA1

(b) What is the purpose of the above sequence of instructions? **(5 marks)**

Q1RU2

____

____

____

This work, IPAT, is an adaptation of a pretest instrument employed in a study "Learning Computer Science Concepts in *Scratch*" by Orni Meerbaum-Salant, Michal Armoni and Moti Ben-Ari, available at http://stwww.weizmann.ac.il/g-cs/*Scratch*/tests-cs-concepts-in-*Scratch*.zip **used under CC BY.**.

THE IMPACT OF *SCRATCH* ON THE ACHIEVEMENTS OF FIRST-YEAR COMPUTER SCIENCE STUDENTS IN SOME NIGERIAN POLYTECHNICS

Introduction to Programming Achievement Test (IPAT) Rubric

INFORMATION FOR TEST MARKERS:

The taxonomy used in this rubric has three categories: unistructural, multistructural and relational cognitive classes. In addition, each category has three cognitive levels: understanding, applying and creating.

Unistructural cognition means that the student has a local perspective mainly knowing only one item or aspect of body of concepts. The other points or ideas are missed neither can the student make connections between related ideas.

Multi-structural cognition means the student knows or makes use of several ideas or concepts in his or her answer. However, the student fails to make connections between these related ideas.

Relational cognitive category means that the student has knowledge of all the related ideas or concepts and is able to make the appropriate connection among them.

PART 1: TESTING CONCEPTUAL PROGRAMMING KNOWLEDGE

(MU = Multi-structural Understanding). CMU stands for Conceptual Multicultural Understanding.

Here 10 programming concepts are given to the students to measure their learning at the level of multi-structural understanding of these concepts.

For CMU1 – 10, if the students shows:

- Complete and correct understanding – **2 marks**
- Incomplete but correct understanding – **1 marks**
- Incorrect answers – **0 mark**

SUBTOTAL = 20 marks

Concept	Explanation (2 marks each)	For office use Only
Program	A program is a set of instructions that are executed by a computing device in order to perform a task or solve a problem. Programs are codes or routines or applications written in a particular programming language that can be understood or translated, and then executed by a computing device.	CMU1
Algorithm	An algorithm is a finite ordered list of steps for solving a computational problem or performing a task.	CMU2
Assignment	Assignment is an operation or a statement in a program that assigns the result (or value) of an expression to a variable.	CMU3
Output	This is the result of a program that may be displayed or written on the monitor as a soft copy or on paper by a printer as a hard copy.	CMU4
	Information resulting from processing input to a computing device.	

Variable	An identifier in a program that can assume different values. A placeholder whose values can change during the execution of a program.	CMU5
Input	Data entered into a computing device.	CMU6
Looping structure	A set of commands or statements in a program to be repeatedly executed by a computing device.	CMU7
Selection structure	A block of statements in a program that makes a computing device to take alternative execution path depending on specific condition.	CMU8
Sequence structure	A block of statements that are to be executed in a serial manner. That is, the execution of statements are performed one after another strictly in the order they are placed in a sequence.	CMU9

	Arithmetic operators are symbols in an expression in a program that indicate arithmetic operations to be performed during the execution of the program.	

PART 2: TESTING COMPUTATIONAL/PROGRAMMING KNOWLEDGE

Question 1:

- **Q1MA1** (MA = Multi-structural Applying). What is tested here is almost like code-tracing where the student is expected to reason following steps in a piece of code in order to arrive at a particular result.

Following the given algorithm, we have the changes in the positions of the cards as shown below:

Since 24 > 2, then we need to swap the positions of the cards

24	2	15

Since 24 > 15, then we need to swap the positions of the cards

2	24	15

2½ marks

2	15	24

2½ marks

Now the cards are sorted with no card on the left having a number greater than the number on the card at the right.

- **Q1RU1** – (RU = Relational Understanding)> What it is tested here is code explaining ability of the student.

  Answer:

  The purpose of the instructions (or the given algorithm) is to rearrange (or sort) the cards (or numbers) in ascending order.

5 marks

SUBTOTAL = 10 marks

Question 2:

Q2MA2 (MA = Multi-structural Applying). What is tested here is code-tracing skill where the student is expected to reason following steps in a piece of code in order to arrive at a particular result.

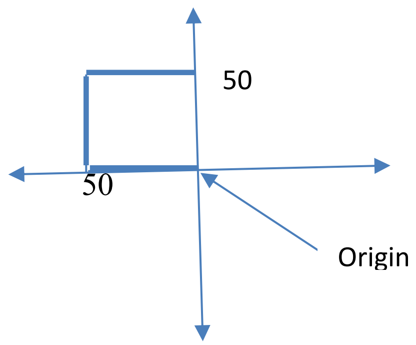

Marks break down:

1 mark – for identifying the origin and starting at the right position.

½ mark for turning (tracing the path) in the right direction (i.e. to the left)

1 mark for carrying out the loop and arriving at the right point (50,0) i.e.50 units on the x axis.

½ mark for turning (tracing the path) in the right direction (i.e. to the right)

1 mark for carrying out the loop and arriving at the right point (50,50) i.e. 50 units and 50 units on the x axis and y axis respectively

½ mark for turning (tracing the path) in the right direction (i.e. to the right)

1 mark for carrying out the loop and arriving at the right point (0, 50) i.e. 50 units on the y axis.

1 mark for identifying the path as the third letter of the alphabet (Letter C)

Q2MC2 (MC = Multi-structural Creating) What is tested here is similar to code tracing, but actually it is code-writing skill we want to measure here.

The three instructions to be added to make the path traced look like a square are:

8. Turn right	**- 1 mark**
9. Carryout step 9.1 10 times	**-1½ marks**
9.1 Move 5 steps	**- 1 mark**

SUBTOTAL = 10 marks

Question 3:

Q3UU1 (UU = Unistructural Understanding)

Answer: Dayo **- 1 mark**

Q3UU2

Answer: Ada **- 1 mark**

Q3MA3. (Code tracing skill)

Answer: 10 minutes. Since Dayo's turn comes after those of Uche and Musa, and each of these two forerunners will run for five minutes.

(1½ marks)

Q3MA4. (Code tracing skill)

Answer: 50 minutes. There are 10 members in this group with each running for 5 minutes. **(1½ marks)**

Q3MA5 (Code tracing skill)

Answer: 70 minutes. There are 10 members in this group with each running for 7 minutes. **(1½ marks)**

Q3RA1. (The way the race runs is like the way a code works. So, we want to test whether the student understands this way. So, it is a code-explaining skill being measured here)

Answer: 70 minutes. The race starts at the same time for the two competing groups (i.e., concurrently). When the first team has finished the race, the eight member of the second team is just starting to run. So the whole race ends when the last member of the second team gets back to the starting line. **(2 marks)**

Q3RA2 (The way the race runs is like the way a code works. So, we want to test whether the student understands this way. Hence it is a code-explaining skill being measured here)

Answer: The first group will take longer than 50 minutes to complete the race and the second group may win the race. **(1½ marks)**

SUBTOTAL = 10 marks

TOTAL FOR THE TEST = 50 marks
