# Supplementary material for: In search of experimental evidence on Scratch programming and students’ achievements in the first-year college computing class? Consider these datasets
Source: Data Brief. 2022 Sep 24;45:108635. doi: 10.1016/j.dib.2022.108635 (PMC9679459; doi:10.1016/j.dib.2022.108635)
Supplement: Supplementary file 2 [file mmc2.pdf]

Impact of *Scratch* on the Achievements of First-year Computer Science Students in Programming in some Nigerian Polytechnics

INFORMED CONSENT FORM

Purpose of research

The proposed research with the above title is undertaken to investigate what impact *Scratch*, a visual programming environment, can have on the programming ability of first-year computer science students. Likely outcomes are significantly positive, none or significantly negative effects on students' programming ability and attitude.

Risks and benefits

No other risk is involved in this research other than normal risks of participating in classes as a student that you are used to. Potential benefits of the study include providing scientific knowledge about the use of *Scratch* in introductory programming class. If the result provides positive evidence in favour of introducing first-year undergraduate students to programming using *Scratch*, this can lead to improved teaching and learning for students.

This research requires your answering voluntarily questions in questionnaire, achievement test and possibly interview at the end of the six weeks of classes. Data to be collected from you include demographic information about yourself, educational and programming background, as well as knowledge, experiences and opinions about programming in the class you will participate in.

Methods of study and participants' actual role in research

The research will make use of the following research methods: questionnaire, achievement test, observation of class sessions and semi-structured interview. The questionnaires which will be administered once in class at the beginning of the study will take 20 minutes while the achievements tests (which will be taken twice, i.e., before the programming class and after six weeks of instruction) will take about 60 minutes. The interview to be taken by selected participants will last for about 45 minutes.

Identity of the researchers

In case you have any question about the research, you are free to contact:

Oladele Campbell (the researcher)–ISTE, University of South Africa. +2348059062424
Prof H.I. Atagana (Supervisor)–ISTE, University of South Africa. +27822009855

Why were you selected?

The method used for selecting the participants in this study is multistage. First, we have used purposive sampling to select four federal polytechnics among the thirteen accredited polytechnics running National Diploma programme in computer science in the north central region. Second, the same earlier sampling technique was used to assign your class to one of the two study groups. You have been selected for this study (after due permission from your school authority) because the study involves students in their first-year computer science programme in selected Nigerian polytechnics.

Privacy, anonymity, and confidentiality

I assured you that we will respect your right to privacy in during and after this research. Data to be collected will be used only for the research. Information that can jeopardize your privacy, anonymity and confidentiality will be removed or replaced by pseudonyms in research reports.

Future use of information.

Information got from this research will be published in my PhD thesis, research paper(s) in conferences and journal, and online repositories for educational and research purposes.

Right not to participate and to withdraw

Please be informed that you have the right to decline from participating in this study or to withdraw from your earlier given consent at any time without fear of any penalty. You are also free to answer or decline from answering certain questions in the questionnaire, achievement test, or the interview. In addition, if you are selected and consent to participate in the interview you are free to object the use of data gathering devices such camera, tape recorder etc.

PARTICIPANT'S CONSENT

I have read the information presented above about the study. I have had the opportunity to ask any questions related to this study and I have received satisfactory answers to my questions. I am aware that, if I am selected for interview at the end of the study, I have the option of allowing my interview to be audio recorded to ensure an accurate recording of my responses. I am also aware that information to be collected in the study may be included in publications to come from this research, with the understanding that the Personally Identifiable Information (PII) about me will be made anonymous. I was informed that I may withdraw my consent at any time without penalty. With full knowledge of all foregoing, I agree, of my own free will, to participate in this study.

Participant's Name:____

Participant's Signature:____

Researcher's Name:____

Researcher's Signature:____

Date:____
