# Supplementary figures and images for: In search of experimental evidence on Scratch programming and students’ achievements in the first-year college computing class? Consider these datasets
Source: Data Brief. 2022 Sep 24;45:108635. doi: 10.1016/j.dib.2022.108635 (PMC9679459; doi:10.1016/j.dib.2022.108635)

Ethical Clearance, Study Request and Permission Letters

**
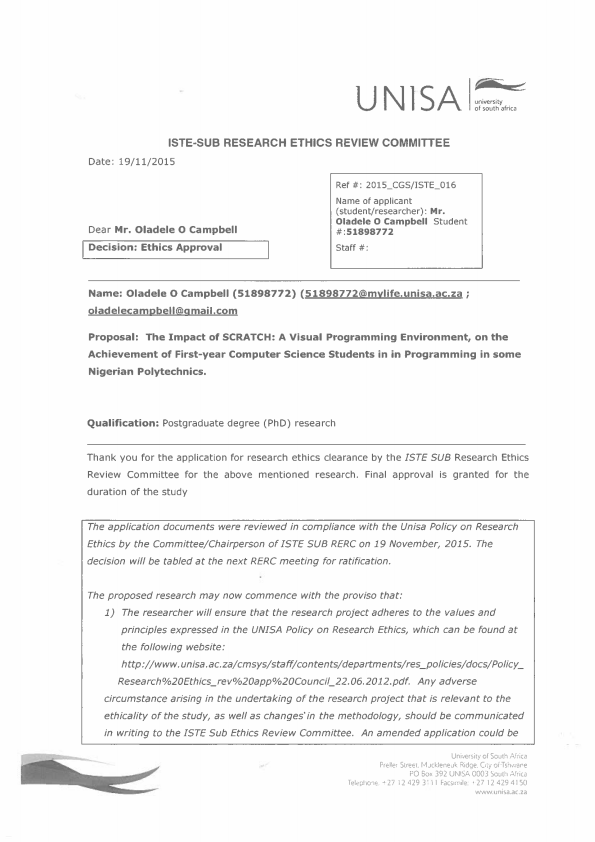
**

**
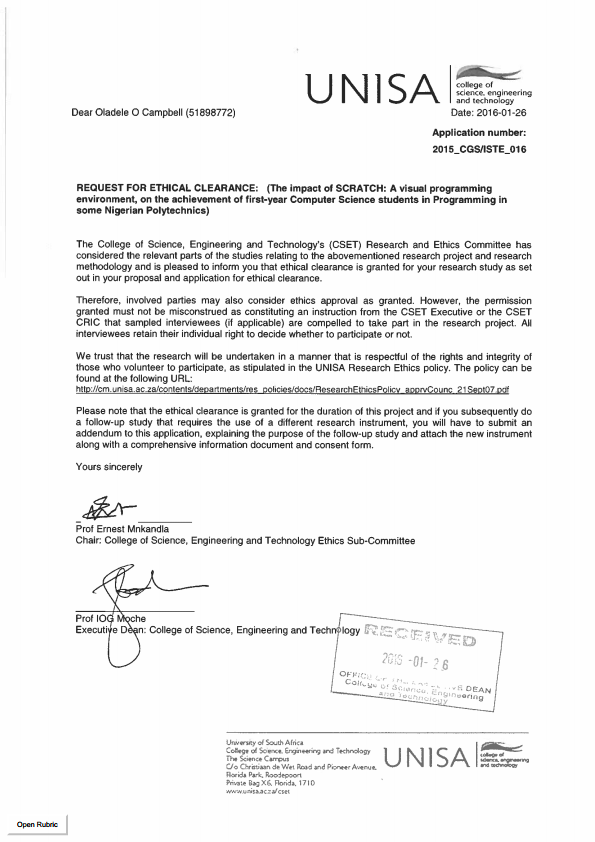
**


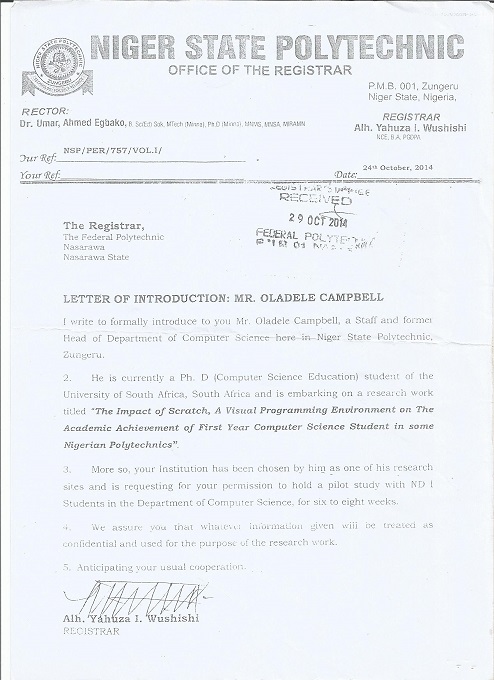


**
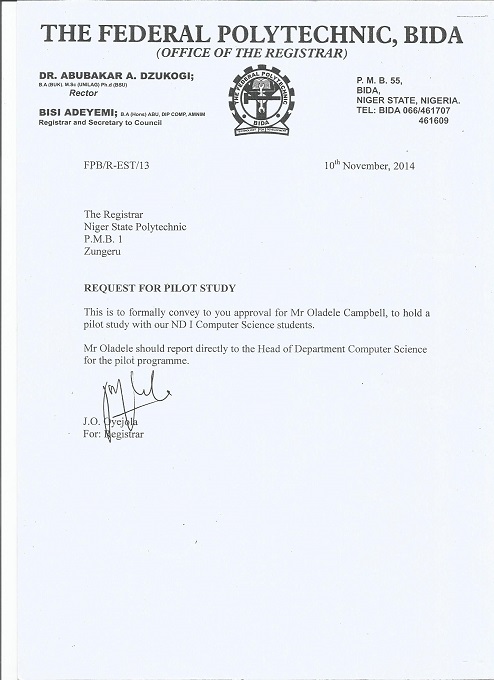
**

**
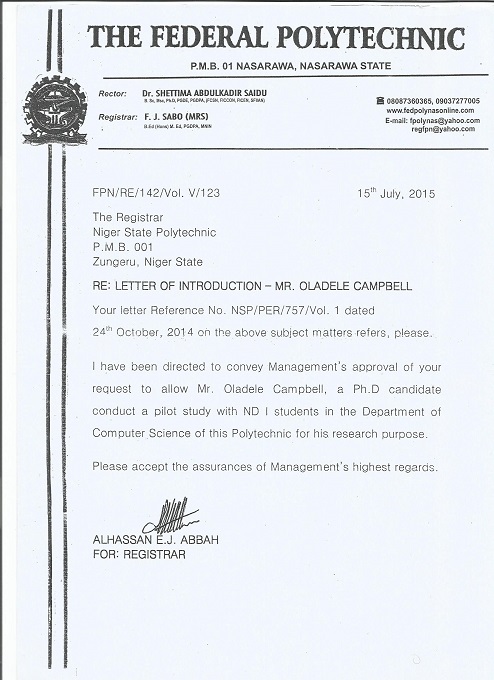
**

**
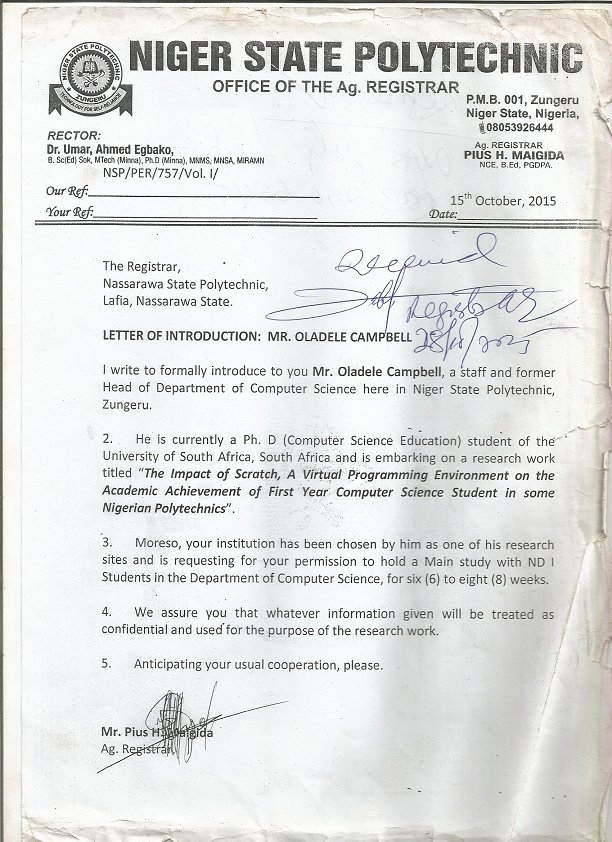
**

**
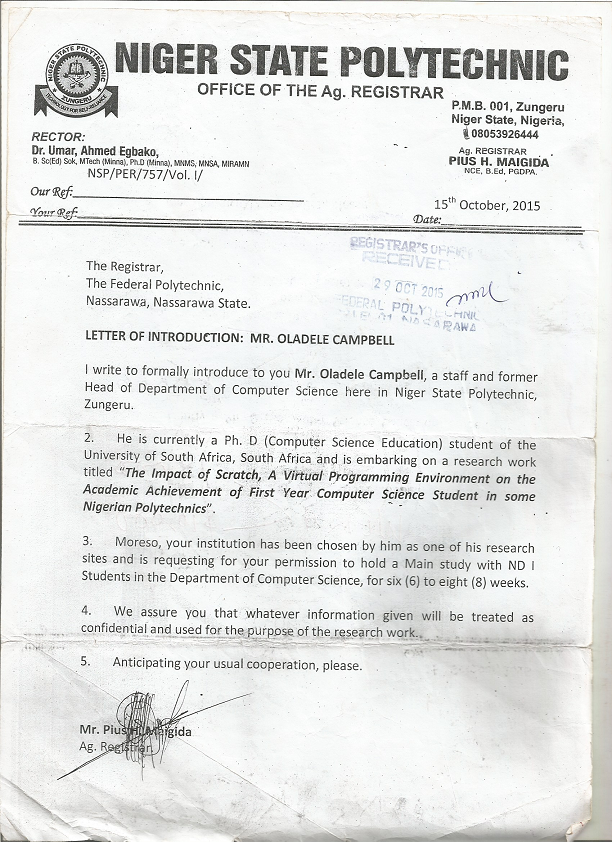
**

Supplement: Supplementary file 3 [file mmc3.docx]
